# Associations between alcohol consumption and anxiety, depression, and health-related quality of life in colorectal cancer survivors

**DOI:** 10.1007/s11764-021-01090-y

**Published:** 2021-09-16

**Authors:** Dóra Révész, Martijn J. L. Bours, Johannes A. Wegdam, Eric T. P. Keulen, Stéphanie O. Breukink, Gerrit D. Slooter, F. Jeroen Vogelaar, Matty P. Weijenberg, Floortje Mols

**Affiliations:** 1 Center of Research on Psychological disorders and Somatic diseases, Tilburg University, PO Box 90153, 5000 LE Tilburg, The Netherlands; 2 Department of Epidemiology, GROW School for Oncology and Developmental Biology, Maastricht University, Maastricht, The Netherlands; 3 Department of Surgery, Elkerliek Hospital, Helmond, The Netherlands; 4 Department of Internal Medicine and Gastroenterology, Zuyderland Medical Centre, Sittard-Geleen, The Netherlands; 5 Department of Research, Netherlands Comprehensive Cancer Organization (IKNL), Utrecht, The Netherlands; 6 Department of Surgery, Maastricht University Medical Centre, Maastricht, The Netherlands; 7 Nutrim School of Nutrition and Translational Research in Metabolism, Maastricht University, Maastricht, The Netherlands; 8 Departments of Surgery and Oncology, Máxima Medical Center, Veldhoven, The Netherlands; 9 Departments of Surgery, VieCuri Medical Center, Venlo, The Netherlands

**Keywords:** Alcohol drinking, Anxiety, Cancer survivors, Colorectal cancer, Depression, Oncology, Quality of life

## Abstract

**Purpose:**

Alcohol consumption is a major risk factor for colorectal cancer (CRC). It is currently poorly understood, however, how alcohol and different alcoholic beverage types are related to psychosocial outcomes in CRC survivors.

**Methods:**

We used data of *N* = 910 CRC survivors from the pooled EnCoRe and PROCORE cohorts and harmonized them into five time points: at diagnosis and 3, 6, 12, and 24 months post-diagnosis. Generalized estimated equation models were used to examine longitudinal associations of alcohol consumption, including consumption of beer, wine, and liquor, with anxiety, depression, and health-related quality of life (HRQoL), while correcting for sociodemographic, lifestyle, and clinical factors.

**Results:**

Survivors were on average 67 years and 37% was female. In the first 2 years post-diagnosis, survivors who consumed more alcoholic drinks/week reported lower anxiety and depressive symptoms and better HRQoL on all domains and symptom scales. This was the case for moderate and heavy amounts of alcohol and mostly for consuming beer and wine, but not for liquor. Associations were more often significant for men and for younger persons (< 67 years at baseline).

**Conclusions:**

Generally, alcohol consumption was observed to be longitudinally related to less anxiety and depression and better HRQoL in CRC survivors.

**Implications for Cancer Survivors:**

Although alcohol consumption is generally unfavorable due to increased risk of carcinogenesis and worse prognosis after CRC, it seems to be associated with better psychosocial outcomes in the first 2 years after diagnosis and treatment. More research is needed to gain knowledge about reasons for drinking and causality.

**Supplementary Information:**

The online version contains supplementary material available at 10.1007/s11764-021-01090-y.

## Background

Colorectal cancer (CRC) is the second most common cancer and cause of cancer death in Europe [1], and the number of CRC survivors is increasing [2]. Alcohol consumption is a major risk factor for CRC. The authoritative report of the World Cancer Research Fund/American Institute for Cancer Research (WCRF/AICR) on lifestyle and cancer prevention states that all alcohol is disadvantageous and there is no difference in cancer risk between beer, wine, and liquor [3]. Moreover, the WRCF/AICR report recommends limiting alcohol consumption after a cancer diagnosis because of adverse effects on cancer prognosis and risk of other diseases (e.g., cardiovascular disease) [3]. However, findings on how alcohol consumption, including specific alcoholic beverage types, is related to psychosocial outcomes after a cancer diagnosis are inconsistent at present.

In cancer survivors, psychosocial problems are prevalent in roughly one third of the population, and these complaints often persist years after treatment [4]. Psychosocial problems are not only associated with various late effects of cancer, but they have also been shown to have a negative impact on morbidity and mortality [5] and might be a barrier for behavioral changes [6]. Alcohol consumption is also suggested to play a role in reduced psychosocial wellbeing, such as lower health-related quality of life (HRQoL) in head and neck squamous cell carcinoma patients [7]. Cross-sectional studies have observed associations between hazardous alcohol use and major depressive episodes in head and neck cancer patients [8] and between alcohol problems and clinical anxiety in testicular cancer patients [9]. Additionally, in 155 CRC survivors 2–10 years post-diagnosis, a less healthy lifestyle (i.e., high alcohol use, low physical activity level, unhealthy dietary habits, high BMI, and current smoking) was cross-sectionally associated with worse physical functioning and more fatigue, but not with depression or anxiety, and there were no separate analyses of alcohol consumption [10]. Another study, which was longitudinal, investigated female CRC survivors up to 20 years post-diagnosis and found that anxiety, more than depression, was related to a less healthy lifestyle (including alcohol) during their follow-up [11]. However, in these female survivors, lower alcohol consumption was reported among women with more anxiety or depression in the first 8 years post-diagnosis [11]. Generally, alcohol is believed to reduce negative and enhance positive emotions, to emphasize feelings of relaxation, and to increase social bonding [12].

Nevertheless, not all studies have found an association between alcohol and psychosocial outcomes. One study found that depression in advanced gastrointestinal cancers was mainly related to tobacco use instead of alcohol consumption and that tobacco smokers were more emotionally affected by their disease [13]. Furthermore, in a randomized psychosocial counseling intervention study among 204 cervical cancer patients, no associations were observed between baseline (9–30 months post-diagnosis) alcohol consumption and quality of life, depression, anxiety, or distress, only an association between patients’ general lifestyle (including physical activity, smoking, and alcohol consumption) and these psychosocial outcomes [14].

Overall, most of the previous research was cross-sectional, performed in survivors with other cancer types than CRC or long after diagnosis. No longitudinal studies have been performed on the relationship over time between alcohol use and psychosocial outcomes immediately after CRC diagnosis and treatment. Moreover, none of the previous studies has shed light on the role of different alcoholic beverage types. Therefore, we aimed to assess longitudinal associations of alcohol consumption (both total alcohol and beer, wine, and liquor) with anxiety, depression, and HRQoL as psychosocial outcomes in CRC survivors prospectively followed up from diagnosis until ~ 2 years post-diagnosis, using pooled data from the Dutch EnCoRe and PROCORE cohorts.

## Methods

### Setting and participants

We used data from the EnCoRe and PROCORE cohorts, which are two ongoing multi-center observational studies including CRC survivors. We pooled the datasets and harmonized the time points as shown in Supplemental Fig. 1. The non-response rate at follow-up measurements is relatively low (< 10% for EnCoRe and 21% for PROCORE).

#### The “energy for life after colorectal cancer” (EnCoRe) study

EnCoRe is an ongoing multi-center prospective cohort study for which adult stage I–III CRC patients are enrolled at diagnosis and followed up at 6 weeks, 6 months, and 1 and 2 years after treatment [15]. These follow-ups corresponded to approximately 3, 6, 12, and 24 months after diagnosis. For the present analyses, we used data collected from April 2012 till July 2018. Patients are recruited at three hospitals in the south-east of the Netherlands. The study has been registered at the Netherlands Trial Registry (www.trialregister.nl, NL6904) and was approved by the Medical Ethics Committee of the University Hospital Maastricht and Maastricht University (number NL38786.068.11) in the Netherlands. In total, we used data of *N* = 445 participants available with data on alcohol factors at baseline.

#### The “patient reported outcomes colorectal cancer” (PROCORE) study

The PROCORE study, which started in 2016, is a prospective population-based study, in which newly diagnosed CRC survivors are recruited after diagnosis and followed up until 12 and 24 months post-diagnosis via the PROFILES registry (Patient Reported Outcomes Following Initial treatment and Long term Evaluation of Survivorship) [16]. Ethical approval for the study was obtained from the certified Medical Ethics Committee of Medical Research Ethics Committees United (number NL51119.060.14). All respondents gave informed consent. Respondents were recruited from four Dutch hospitals, and inclusion criteria were the diagnosis of CRC stage I–IV and being 18 years or older. For this specific paper, data were used of *N* = 465 participants that had entered their alcohol consumption at baseline.

### Psychosocial outcomes

Anxiety and depressive symptom scores were calculated from the Hospital Anxiety and Depression Scale (HADS), which consists of seven items for anxiety and seven for depression (range 0–21 points), with higher scores indicating more symptoms [17]. A cut-off value of 8 points for each subscale was used to identify a clinically relevant level of anxiety or depressive symptoms [18].

HRQoL was assessed with the cancer-specific European Organization for the Research and Treatment of Cancer Quality of Life Questionnaire—Core 30 (EORTC QLQ-C30, version 3.0) [19]. For the present analyses, we used the HRQoL domains global quality of life and cognitive, emotional, physical, role, and social functioning and the symptom scales fatigue, pain, and nausea/vomiting. According to the EORTC guidelines, a sum score was calculated for every domain and symptom scale (0–100 points). Higher scores on domains represent better quality of life or functioning, whereas higher scores on symptom scales reflect more complaints [19].

### Alcohol consumption

In EnCoRe, habitual alcohol consumption in the year before CRC diagnosis was assessed at diagnosis with a Food Frequency Questionnaire (FFQ). During follow-up, 7-day food diaries were used to assess alcohol consumption and intake of specific alcoholic beverages over the past week [20]. The validity of the FFQ has been evaluated within EnCoRe relative to the 7-day food diaries, showing that the intake of alcohol was highly correlated between both methods (rho = 0.91) [21]. In PROCORE, alcohol consumption was recorded at every time point with questions about the average frequency of alcohol consumption per week in the past year and the number of glasses of beer, wine, and liquor.

Alcohol consumption was defined as (A) drinking alcohol (y/n); (B) the number of drinks per week; (C) drinking beer, wine, or liquor; (D) the number of beer, wine, or liquor drinks per week; and (E) the categories of non-drinkers, moderate (< 14 drinks/week), and heavy drinkers (≥ 14 drinks/week) [22]. For each alcoholic drink, we assumed that all types of alcoholic beverages, i.e., beer, wine, or liquor, contain 10 g ethanol per unit of consumption [22].

### Covariates

Information was collected about age and sex, and education level, which was categorized as low (lower vocational and primary education), medium (intermediate vocational and secondary education), and high level (higher vocational and university). Physical activity was calculated in hours per week of moderate-to-vigorous physical activities (MVPA) by the Short QUestionnaire to ASsess Health-enhancing physical activity [23]. Smoking was recorded as non-smoker, former, or current smoker. Body mass index (kg/m^2^) was determined based on body height and weight, which were self-reported in PROCORE and measured by research dietitians during patient visits in EnCoRe. The Self-Administered Comorbidity Questionnaire was used for assessing the number of comorbidities (none, one, or two or more comorbid conditions) [24]. Both studies also recorded tumor localization (colon/rectum), tumor stage (I–IV), treatments received besides surgery (chemotherapy/radiotherapy), and the placing of a stoma (yes/no).

### Statistical analyses

All variables were described as percentages or means and standard deviations or as medians and interquartile ranges for non-normally distributed factors. We compared baseline characteristics of survivors with or without alcohol consumption using chi-square tests for categorical variables and independent sample *t*-tests and Mann–Whitney *U* tests for normally or non-normally distributed continuous variables, respectively.

Next, in order to determine associations of alcohol consumption and beverage types with psychosocial outcomes over time, we used generalized estimating equations (GEE) with an exchangeable correlation structure. GEE analyses take into account within-person correlations when examining multiple observations per subject over time and it can handle missing subjects [25]. First, we analyzed associations between the number of drinks of alcohol and psychosocial outcomes with linear GEE and with logistic GEE for dichotomous clinical anxiety and depression. Subsequently, we analyzed the categories of drinking as determinant, by adding dummy variables for moderate and heavy drinkers, to see if the relation between alcohol and psychosocial outcomes depends on dosage. We corrected analyses for cohort, age, sex, education, physical activity, smoking status, BMI, months since diagnosis, cancer localization, chemotherapy, radiotherapy, and stoma placement.

We also analyzed the associations between drinks per week and psychosocial outcomes in the drinkers only, in order to determine whether the non-drinkers could have influenced results. Additionally, we ran the analyses for the separate beverage types mutually adjusted for the other beverage types, in order to examine independent associations. Next, we stratified the analyses for sex and age in order to explore potential effect modification of the association between alcohol consumption and psychosocial outcomes. Alcohol may have different effects on social factors in males vs. females, as shown in prairie voles [26]. For age stratification, we used the mean age (≤ 67 vs. > 67 years), which was close to the retirement age and may therefore influence the role of drinking in social relationships. Lastly, we analyzed the associations between alcoholic drinks and psychosocial outcomes in both cohorts separately, to confirm that pooling of the data had not altered the results. All analyses were conducted using SPSS version 24.0 (IBM Corp., Armonk, NY, USA). Significance level was set at *p* < 0.01, two-tailed.

## Results

### Sample characteristics

Baseline sample characteristics are shown for the pooled cohorts and compared for the non-drinkers (*N* = 191) vs. drinkers of alcohol (*N* = 719) in Table 1. On average, survivors were 67 years old, 37% was female, and all were approximately 0.6 months after diagnosis at baseline. Alcohol drinkers were slightly younger, less often female, with higher education levels, more physically active, more often former smoker, and had more often received chemotherapy.

**Table 1:** Baseline characteristics of CRC survivors from the pooled EnCoRe and PROCORE cohorts and comparison of non-drinkers and alcohol drinkers

	Total(*N* = 910)	No alcohol(*N* = 191)	Alcohol(*N* = 719)	*p* value^b^
Sociodemographics (*N*(%))				
Age (years, mean(SD))	66.9(9.2)	68.9(9.8)	66.3(8.9)	**0.001**
Sex (females)	332(36.5)	113(59.2)	220(30.6)	** < 0.001**
Education level				
High	270(29.7)	23(12.4)	247(34.4)	** < 0.001**
Medium	556(61.5)	129(69.4)	427(59.5)	
Low	78(8.6)	34(18.3)	44(6.1)	
Lifestyle factors (*N*(%))				
Physical activity (median, IQR)^a^	11(12)	9.2(11.4)	11.0(11.5)	**0.002**
Smoking status				
Never	273(30.0)	78(41.1)	195(27.1)	** < 0.001**
Former	527(58.0)	83(43.7)	444(61.8)	
Current	109(12.0)	29(15.3)	80(11.1)	
BMI (kg/m^2^, mean(SD))	27.4(4.4)	27.8(4.8)	27.3(4.3)	0.14
Clinical factors (*N*(%))				
Months since diagnosis (median(range))	0.6(0–11)	0.6(0–6)	0.6(0–11)	0.71
Tumor location				
Colon	604(66.4)	136(71.2)	468(65.4)	0.13
Rectum	303(33.3)	55(28.8)	248(34.6)	
Staging				
I	254(29.0)	54(28.7)	200(29.1)	0.07
II	236(27.0)	64(34.0)	172(25.0)	
III	367(41.9)	67(35.6)	300(43.7)	
IV	18(2.1)	3(1.6)	15(2.2)	
Chemotherapy	325(35.7)	53(27.7)	272(37.8)	**0.01**
Radiotherapy	194(21.3)	35(18.3)	159(22.1)	0.26
Stoma placement	3(0.3)	1(0.5)	2(0.3)	0.59
Number of co-morbidities				
None	120(26.2)	22(17.9)	98(29.3)	0.02
1	157(34.3)	42(34.1)	115(34.3)	
≥ 2	181(39.5)	59(48.0)	122(36.4)	

^a^Hours/week of MVPA (moderate-to-vigorous physical activity)

^b^Independent samples *t*-tests for normally distributed continuous variables; Mann–Whitney *U* tests for not-normally distributed continuous variables; chi-square tests for categorical variables. Significant *p* values < 0.01 are represented bold

Table 2 shows the amount of alcohol consumption and anxiety, depression, and HRQoL scores reported at each time point. The percentage of non-drinkers increased from 21% at diagnosis (baseline) to 30% at 24 months post-diagnosis. Whereas 19–22% of the moderate or heavy drinkers at diagnosis stopped drinking during follow-up, 8–9% of non- or moderate drinkers became heavy drinkers.

**Table 2:** Alcohol consumption and psychosocial outcomes (depression, anxiety, and HRQoL) at each time point

Means (standard deviations) or *N*(%)	After diagnosis	3 m post-diagnosis	6 m post-diagnosis	12 m post-diagnosis	24 m post-diagnosis
Alcohol consumption^a^	*N* = 910	*N* = 383	*N* = 332	*N* = 629	*N* = 396
Total alcohol (drinks/week)	8.1(10.6)	9.2(13.4)	9.5(13.7)	7.7(11.1)	7.9(11.2)
Non-drinker	191(21.0)	122(31.9)	104(31.3)	179(28.9)	118(29.8)
Moderate	512(56.3)	174(45.4)	142(42.8)	318(50.6)	197(49.7)
Heavy	207(22.7)	87(22.7)	86(25.9)	128(20.3)	81(20.5)
Beer (drinks/week)	3.8(8.3)	4.8(11.2)	5.0(11.1)	3.6(8.3)	3.8(9.3)
Non-drinker	450(49.4)	221(57.7)	187(56.3)	383(60.9)	234(59.1)
Moderate	377(41.4)	125(32.6)	110(33.1)	190(30.2)	130(32.8)
Heavy	83(9.1)	37(9.7)	35(10.5)	56(8.9)	32(8.1)
Wine (drinks/week)	3.6(5.8)	3.6(6.1)	3.7(6.3)	3.4(5.7)	3.5(5.5)
Non-drinker	384(42.2)	204(53.3)	176(53.0)	319(50.7)	200(50.5)
Moderate	455(50.0)	147(38.4)	129(38.9)	263(41.8)	167(42.2)
Heavy	71(7.8)	32(8.4)	27(8.1)	47(7.5)	29(7.3)
Liquor (drinks/week)	0.8(2.9)	0.8(3.0)	0.8(2.5)	0.7(2.8)	0.6(2.1)
Non-drinker	679(74.6)	289(75.5)	259(78.0)	514(81.7)	319(80.6)
Moderate	214(23.5)	91(23.8)	69(20.8)	107(17.0)	73(18.4)
Heavy	17(1.9)	3(0.8)	4(1.2)	8(1.3)	4(1.0)
Psychological outcomes^b^	*N* = 467	*N* = 388	*N* = 344	*N* = 637	*N* = 400
Anxiety	5.1(3.9)	3.2(3.2)	3.4(3.3)	3.5(3.5)	3.5(3.7)
Clinical anxiety (HADS cut-off ≥ 8)	116(25.5)	37(9.6)	46(13.6)	78(12.4)	48(12.2)
Depressive	4.0(3.6)	3.5(3.4)	3.6(3.7)	3.4(3.3)	3.6(3.7)
Clinical depression (HADS cut-off ≥ 8)	79(17.4)	53(13.8)	49(14.5)	83(13.2)	56(14.2)
HRQoL domains^b^	*N* = 470	*N* = 389	*N* = 344	*N* = 638	*N* = 400
Global quality of life	75.4(18.4)	75.2(18.2)	76.6(18.4)	78.7(18.0)	78.5(18.9)
Cognitive functioning	89.3(16.2)	86.5(20.2)	85.7(19.4)	87.5(18.5)	85.3(19.9)
Emotional functioning	78.4(20.4)	88.2(17.6)	88.6(16.5)	89.2(17.7)	89.1(16.9)
Physical functioning	89.6(15.1)	77.8(19.4)	82.5(18.0)	85.2(17.7)	85.4(17.4)
Role functioning	85.7(25.2)	72.6(27.4)	81.5(24.0)	84.3(23.4)	85.7(23.7)
Social functioning	88.9(18.8)	83.4(21.2)	89.9(17.9)	90.8(18.1)	90.4(18.8)
HRQoL symptom scales^b^	*N* = 469	*N* = 389	*N* = 344	*N* = 638	*N* = 401
Fatigue	18.7(23.2)	27.6(22.9)	22.4(21.1)	19.0(22.3)	19.2(21.9)
Nausea/vomiting	2.8(8.7)	2.4(8.9)	1.9(7.9)	3.0(11.0)	2.4(8.4)
Pain	10.5(19.7)	17.4(22.8)	13.1(20.3)	12.5(21.6)	13.6(22.2)

^a^We describe categories of alcohol consumption as non-drinkers, moderate (< 14 drinks/week), and heavy drinkers (≥ 14 drinks/week)

^b^Psychological factors and HRQoL measures are measured at baseline for PROCORE only

### Longitudinal associations of alcoholic drinks per week with psychosocial outcomes

Overall, more alcohol consumption was longitudinally associated with lower anxiety and depressive symptoms (Table 3). Alcohol consumption was also associated with better HRQoL on all domains and with less complaints on all symptom scales. Whereas lower anxiety scores over time were mostly related to drinking beer, lower depression scores, higher HRQoL scores, and lower symptom scores were mostly related to drinking wine (Table 3). Drinking liquor was not significantly associated with psychosocial outcomes.

**Table 3:** Longitudinal associations of alcohol consumption (drinks/week) with depression, anxiety, and HRQoL

	Alcohol		Beer		Wine		Liquor	
	B(SE)	*p*	B(SE)	*p*	B(SE)	*p*	B(SE)	*p*
Psychological outcomes (*N* = 814)^a^								
Anxiety	− 0.02(0.01)	**0.001**	− 0.03(0.01)	**0.001**	− 0.03(0.02)	0.09	0.02(0.03)	0.47
Clinical anxiety	− 0.03(0.01)	**0.01**	− 0.03(0.01)	0.06	− 0.03(0.02)	0.06	− 0.00(0.02)	0.87
Depression	− 0.03(0.01)	** < 0.001**	− 0.02(0.01)	0.03	− 0.06(0.01)	** < 0.001**	0.00(0.02)	0.91
Clinical depression	− 0.03(0.01)	0.04	− 0.01(0.01)	0.31	− 0.08(0.02)	**0.001**	− 0.00(0.03)	0.92
HRQoL domains (*N* = 817)^a^								
Global quality of life	0.18(0.04)	** < 0.001**	0.11(0.05)	0.02	0.45(0.08)	** < 0.001**	− 0.16(0.18)	0.37
Cognitive functioning	0.14(0.04)	** < 0.001**	0.13(0.04)	**0.002**	0.20(0.08)	**0.01**	0.02(0.13)	0.90
Emotional functioning	0.11(0.04)	**0.002**	0.09(0.05)	0.06	0.24(0.07)	**0.001**	− 0.09(0.13)	0.50
Physical functioning	0.16(0.03)	** < 0.001**	0.08(0.04)	0.03	0.35(0.07)	** < 0.001**	0.14(0.10)	0.18
Role functioning	0.26(0.05)	** < 0.001**	0.25(0.06)	** < 0.001**	0.43(0.10)	** < 0.001**	− 0.21(0.29)	0.48
Social functioning	0.20(0.04)	** < 0.001**	0.16(0.05)	**0.003**	0.38(0.08)	** < 0.001**	− 0.04(0.13)	0.78
HRQoL symptom scales (*N* = 816)^a^								
Fatigue	− 0.27(0.05)	** < 0.001**	− 0.17(0.05)	**0.002**	− 0.58(0.09)	** < 0.001**	0.00(0.21)	0.99
Nausea/vomiting	− 0.05(0.01)	**0.001**	− 0.03(0.02)	0.14	− 0.10(0.03)	** < 0.001**	− 0.08(0.04)	0.04
Pain	− 0.18(0.05)	**0.001**	− 0.10(0.07)	0.14	− 0.44(0.08)	** < 0.001**	0.05(0.20)	0.82

Corrected for cohort, age, sex, education, physical activity, smoking status, BMI, months since diagnosis, cancer localization, chemotherapy, radiotherapy, and stoma placement. Significant *p* values < 0.01 are represented bold

Analyses in the alcohol consumers only showed similar results (data not shown). Similar results were also observed in analyses of independent associations of the separate beverage types with psychosocial outcomes, when corrected for other beverage types (data not shown).

### Longitudinal associations of alcohol categories with psychosocial outcomes

**Fig. 1:**
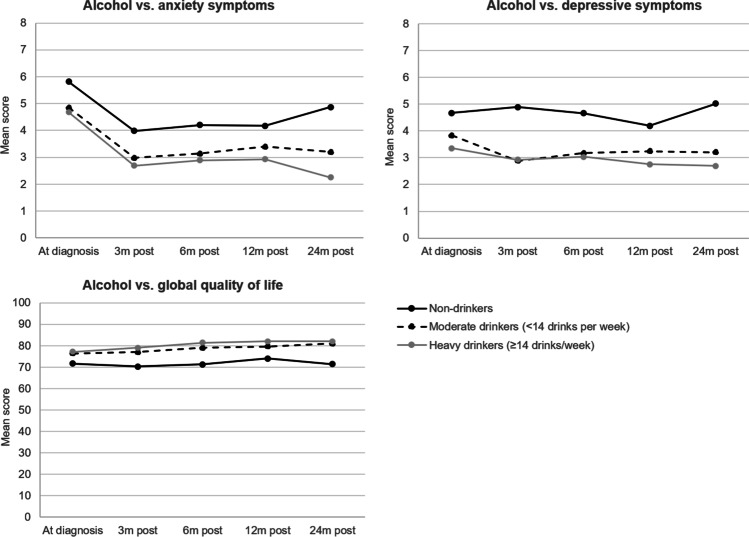
Anxiety and depression, and global quality of life scores are shown at each time point and stratified for alcohol consumption categories (non-drinkers, moderate vs. heavy drinkers)

We only used categories of total alcohol consumption (no/moderate/heavy) in our analyses, because sample sizes for these categories were too low in the beer, wine, or liquor groups, particularly the heavy drinking category. We observed dose–response relationships between alcohol consumption and all psychosocial outcomes (Supplemental Table 1). Relative to non-drinkers, moderate alcohol use was associated with lower anxiety and depression scores over time, while heavy alcohol consumption was associated with even lower depression scores. Survivors reported better HRQoL over time in case of moderate and heavy drinkers, compared to non-drinkers (Supplemental Table 1). Figure 1 shows changes in anxiety and depression scores and global quality of life over time (depicting what happens in most HRQoL domains and symptom scales), stratified for categories of alcohol consumption. At each time point, we saw the largest differences in psychosocial outcomes over time between non-drinkers and drinkers (moderate or heavy), while differences between moderate and heavy drinkers were small. The extended figure including all HRQoL scores is shown in Supplemental Fig. 2.

### Subgroup and sensitivity analyses

Even though betas were in the same direction and often larger in women vs. men, associations were more often significant in the men (Supplemental Table 2). When we stratified for age (≤ 67 years vs. > 67 years), we saw that the associations were often stronger and significant in the group ≤ 67 years (Supplemental Table 3). When we stratified for cohorts, we observed that the betas were similar in both the PROCORE and EnCoRe studies (data not shown).

## Discussion

We examined longitudinal associations of alcohol consumption with anxiety, depression, and HRQoL in colorectal cancer survivors from diagnosis up to 2 years post-diagnosis. Overall, we observed that consuming more alcoholic drinks was related to lower anxiety and depression and to better HRQoL over time, after correction for cohort, months since diagnosis, and sociodemographic, lifestyle, and clinical factors. The associations between alcohol and psychosocial outcomes were only significant for beer and wine consumption, not for liquor consumption. In stratified analyses, significant associations were mostly observed in male and in younger (≤ 67 years) CRC survivors.

Our findings were somewhat unexpected, as various other studies have found the opposite: in patients with various types of cancer, alcohol consumption was shown to be related to more anxiety and depression and lower HRQoL [7–9]. These studies mostly focused on alcohol problems, as defined by high scores on the Alcohol Use Disorders Identification Test and not by the amount of alcoholic drinks calculated per person, which were associated with clinical anxiety and depression [9] or more co-morbidity with depression [8]. One study looked at drinking as a way of coping and found this to be related to lower quality of life [7]. They also measured alcohol consumption and observed relations with lower HRQoL, although their analyses were only corrected for age, sex, neuroticism, and smoking and not for relevant other lifestyle or clinical factors [7].

In addition, some studies have looked at total lifestyle (including alcohol) and not at the separate associations of alcohol with psychosocial outcomes [10, 14]. A study among cervical cancer patients reported that after cancer treatment patients adhering to lifestyle guidelines (i.e., less alcohol, no smoking, more exercise) reported less depression, anxiety, and distress [14]. In their intervention study, patients improved their lifestyle (less alcohol and smoking and more physical activity), although only benefits for quality of life were observed and not for depression, anxiety, or distress [14]. Another study reported that there was no clear association between alcohol and psychosocial outcomes in testicular cancer survivors and found that mostly smoking was related to depressive symptoms [27]. However, this study was between 2 and 10 years post-diagnosis [27], whereas we focused on the first 2 years post-diagnosis. A study among head and neck cancer patients reported that there was a large overlap in depression, alcohol consumption, and smoking [8]. Unfortunately, they did not analyze exact associations between drinking rate and depression scores [8].

Despite the above contradictory results, findings from other research [11, 28, 29] are in line with our findings. In a longitudinal study among female CRC survivors, lower alcohol consumption in women was associated with more anxiety or depression in the first 8 years post-diagnosis [11]. Additionally, among individuals aged 55 to 80 years with type 2 diabetes mellitus, moderate alcohol intake (specifically wine) was significantly associated with lower risk of depression relative to alcohol abstainers [28]. A recent systematic review also reported that lower alcohol intake was related to chemotherapy-related nausea and vomiting in several cancer types (including CRC) [29].

### Clinical implications

Potentially, consumption of alcohol can reduce tension, offering a way of coping with life’s demands and increasing social bonding [12]. In some individuals, alcohol use may elicit either positive emotional reactions (e.g., social bonding) or negative reactions (e.g., social anxiety) [12]. Overall, our findings could be a reflection of survivors slowly regaining their habitual lifestyle habits when things get back to normal after the cancer, including increased participation in social life that may be accompanied by more casual alcohol use (social drinking) and a general sense of feeling better. Moreover, survivors of cancer may express higher gratitude and appreciation for life, and they perhaps view some positive aspects of their cancer diagnosis [30]. Anxiety, depression, and HRQoL are multifactorial constructs, and our findings suggest that alcohol consumption could be one of the factors contributing to these psychosocial outcomes. Future research is needed to gain more knowledge about the specific reasons for consuming alcohol, e.g., whether survivors drink as a coping mechanism or as a social activity. Since we saw more associations between consumption of beer or wine and better psychosocial outcomes, we hypothesize that these beverages are perhaps consumed more often as social activities, while liquor might be consumed more often as a coping mechanism.

### Study limitations

Potential limitations of this study are that we did not measure anxiety, depression, and HRQoL pre-diagnosis. Moreover, we cannot differentiate between former drinkers that may have more depressive symptoms than lifetime abstainers [31]. Even though our study was longitudinal, we cannot conclude anything about causality due to its observational character. Additionally, we pooled and harmonized two studies that had slightly different time points at follow-up after diagnosis. Therefore, we corrected all analyses for months since diagnosis and cohort. Another limitation was that all lifestyle factors were self-reported, and we cannot exclude the possibility that participants answered questions in a socially desirable manner. Graham et al. have also reported that there was a difference in the interpretations of findings on the relationship between alcohol and depression, based on the measurement of alcohol or psychosocial factors and sex [31]. They state that the relationship is often stronger for women vs. men, when depression is measured as a clinical diagnosis instead of depressive symptoms, or when alcohol use is measured as the quantity that persons drink per occasion vs. the frequency of drinking, the total volume and quantity, or the number of heavy drinkers [31]. The strengths of this study were its size with two pooled longitudinal studies that measured CRC survivors repeatedly from diagnosis until 2 years post-diagnosis. We also have data on beverage type consumption at each time point.

## Conclusions

To conclude, this is the first study in CRC survivors that analyzed the longitudinal relation of alcohol consumption with anxiety and depressive symptoms and HRQoL in the first years after diagnosis. More alcohol consumption over time was longitudinally related to less anxiety and depressive symptoms and better HRQoL. Based on our observational data, we cannot rule out the possibility that former drinkers may be more affected by both physical and psychological issues or that there are other factors playing an important role in this association, nor can we draw firm conclusions about causality. Notwithstanding the fact that it is well established that alcohol has many unfavorable effects both in cancer patients and in healthy individuals, our results show that alcohol consumption seems to be associated with better psychosocial outcomes in the first 2 years after CRC diagnosis and treatment. More research is needed to gain more insight into the reasons for alcohol use and potential causality.

## Supplementary Information

Below is the link to the electronic supplementary material.

Supplementary file1 (DOCX 26 KB)

Supplementary file2 (DOCX 430 KB)

## Data Availability

The data that support the findings of this study are available on request from the corresponding author. The data are not publicly available due to privacy or ethical restrictions.
