# Supplementary material for: Associations between alcohol consumption and anxiety, depression, and health-related quality of life in colorectal cancer survivors
Source: J Cancer Surviv. 2021 Sep 16;16(5):988–97. doi: 10.1007/s11764-021-01090-y (PMC9489554; doi:10.1007/s11764-021-01090-y)
Supplement: Supplementary file 1 — Supplementary file1 (DOCX 26 KB) [file 11764_2021_1090_MOESM1_ESM.docx]

| **Supplemental Table 1: Longitudinal associations of categories of alcohol consumption (determinant) with depression, anxiety and HRQoL (outcomes) from diagnosis up to 2 years post-diagnosis in colorectal cancer survivors from the pooled EnCoRe and PROCORE cohorts** | | | | |
| --- | --- | --- | --- | --- |
|  | **Moderate vs. non-drinker** | | **Heavy vs. non-drinker** | |
|  | **B (SE)** | **p** | **B (SE)** | **p** |
| **Psychological factors** | | | | |
| Anxiety score | -0.51 (0.20) | **.01** | -0.59 (0.26) | .02 |
| Clinical anxiety | -0.38 (0.16) | .02 | -0.48 (0.23) | .04 |
| Depression score | -0.87 (0.23) | **<.001** | -1.15 (0.27) | **<.001** |
| Clinical depression | -0.58 (0.18) | **.001** | -0.94 (0.23) | **<.001** |
| **HRQoL domains** | | | | |
| Global quality of life | 4.10 (1.19) | **.001** | 6.28 (1.43) | **<.001** |
| Cognitive functioning | 4.51 (1.29) | **<.001** | 5.02 (1.44) | **<.001** |
| Emotional functioning | 3.09 (1.11) | **.01** | 3.24 (1.39) | .02 |
| Physical functioning | 5.20 (1.02) | **<.001** | 7.44 (1.17) | **<.001** |
| Role functioning | 6.89 (1.59) | **<.001** | 10.38 (1.88) | **<.001** |
| Social functioning | 4.48 (1.25) | **<.001** | 7.94 (1.47) | **<.001** |
| **HRQoL symptom scales** | | | | |
| Fatigue | -6.34 (1.45) | **<.001** | -9.88 (1.83) | **<.001** |
| Nausea / vomiting | -2.08 (0.66) | **.002** | -2.73 (0.70) | **<.001** |
| Pain | -5.21 (1.46) | **<.001** | -8.31 (1.75) | **<.001** |
| **Footnotes**: Corrected for cohort, age, sex, education, physical activity, smoking status, BMI, months since diagnosis, cancer localization, chemotherapy, radiotherapy and stoma placement. a) We describe the categories of alcohol consumption as non-drinkers, moderate drinkers (<14 drinks/week) and heavy drinkers (≥14 drinks/week). Significant p-values<0.01 are represented bold; | | | | |

| **Supplemental Table 2: Linear GEE models of longitudinal associations between alcoholic drinks/week and psychosocial outcomes, stratified for men vs. women** | | | | |
| --- | --- | --- | --- | --- |
|  | **Men (N=526)** | | **Women (N=288)** | |
|  | **B (SE)** | **p** | **B (SE)** | **p** |
| **Psychological factors** |  | |  | |
| Anxiety score | -0.02 (0.01) | **.004** | -0.02 (0.03) | .38 |
| Anxiety cut-off | -0.02 (0.01) | .05 | -0.05 (0.02) | .06 |
| Depression score | -0.02 (0.01) | **.002** | -0.06 (0.02) | .02 |
| Depression cut-off | -0.02 (0.01) | .08 | -0.11 (0.05) | .04 |
| **HRQoL domains** |  | |  | |
| Global quality of life | 0.16 (0.04) | **<.001** | 0.35 (0.14) | **.01** |
| Cognitive functioning | 0.13 (0.04) | **.001** | 0.19 (0.13) | .15 |
| Emotional functioning | 0.10 (0.04) | **.01** | 0.13 (0.14) | .36 |
| Physical functioning | 0.13 (0.04) | **<.001** | 0.37 (0.11) | **.001** |
| Role functioning | 0.23 (0.05) | **<.001** | 0.51 (0.18) | **.01** |
| Social functioning | 0.18 (0.04) | **<.001** | 0.29 (0.14) | .04 |
| **HRQoL symptom scales** |  | |  | |
| Fatigue | -0.25 (0.05) | **<.001** | -0.35 (0.18) | .06 |
| Nausea / vomiting | -0.04 (0.01) | **.003** | -0.13 (0.06) | .03 |
| Pain | -0.14 (0.05) | **.01** | -0.36 (0.16) | .03 |
| **Footnotes**: GEE analyses are corrected for cohort, age, education, physical activity, smoking status, BMI, months since diagnosis, cancer localization, chemotherapy, radiotherapy and stoma placement. Significant p-values<0.01 are represented bold. | | | | |

| **Supplemental Table 3: Longitudinal associations between alcoholic drinks/week and psychosocial outcomes, stratified for survivors ≤67 years vs. >67 years of age** | | | | |
| --- | --- | --- | --- | --- |
|  | **≤67 years (N=428)** | | **>67 years (N=386)** | |
|  | **B (SE)** | **p** | **B (SE)** | **p** |
| **Psychological factors** |  | |  | |
| Anxiety score | -0.03 (0.01) | **.01** | -0.01 (0.01) | .43 |
| Anxiety cut-off | -0.04 (0.01) | **.01** | -0.01 (0.01) | .56 |
| Depression score | -0.03 (0.01) | **.002** | -0.01 (0.01) | .27 |
| Depression cut-off | -0.03 (0.01) | **.02** | -0.02 (0.02) | .47 |
| **HRQoL domains** |  | |  | |
| Global quality of life | 0.22 (0.06) | **<.001** | 0.11 (0.07) | .09 |
| Cognitive functioning | 0.19 (0.05) | **<.001** | 0.05 (0.05) | .35 |
| Emotional functioning | 0.17 (0.05) | **.001** | 0.01 (0.05) | .88 |
| Physical functioning | 0.17 (0.05) | **.001** | 0.15 (0.05) | **.002** |
| Role functioning | 0.32 (0.07) | **<.001** | 0.18 (0.08) | .03 |
| Social functioning | 0.22 (0.06) | **<.001** | 0.13 (0.05) | .01 |
| **HRQoL symptom scales** |  | |  | |
| Fatigue | -0.29 (0.07) | **<.001** | -0.22 (0.07) | **.002** |
| Nausea / vomiting | -0.08 (0.02) | **<.001** | -0.01 (0.02) | .53 |
| Pain | -0.22 (0.08) | **.004** | -0.10 (0.07) | .16 |
| **Footnotes**: GEE analyses are corrected for cohort, sex, education, physical activity, smoking status, BMI, months since diagnosis, cancer localization, chemotherapy, radiotherapy and stoma placement. Significant p-values < 0.01 are represented bold. | | | | |
