# Supplementary material for: Associations between alcohol consumption and anxiety, depression, and health-related quality of life in colorectal cancer survivors
Source: J Cancer Surviv. 2021 Sep 16;16(5):988–97. doi: 10.1007/s11764-021-01090-y (PMC9489554; doi:10.1007/s11764-021-01090-y)
Supplement: Supplementary file 2 — Supplementary file2 (DOCX 430 KB) [file 11764_2021_1090_MOESM2_ESM.docx]

***
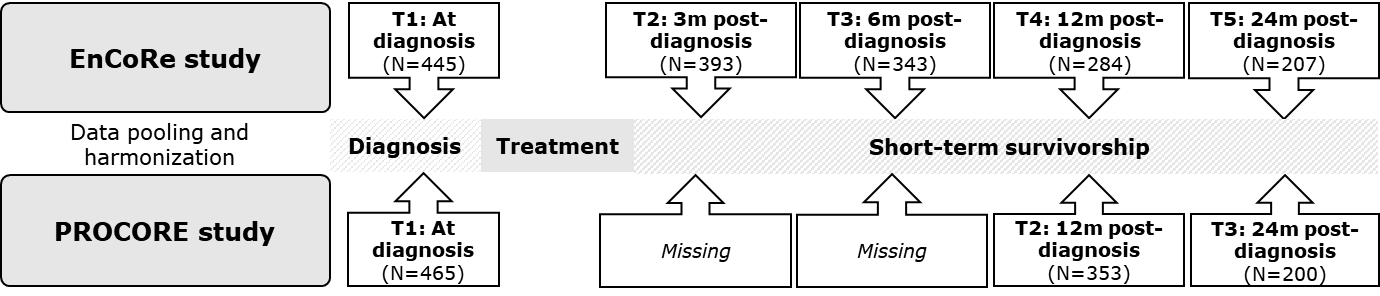
***

***Supplemental Figure 1****: Schematic representation of the two datasets and how they are pooled and harmonized.*


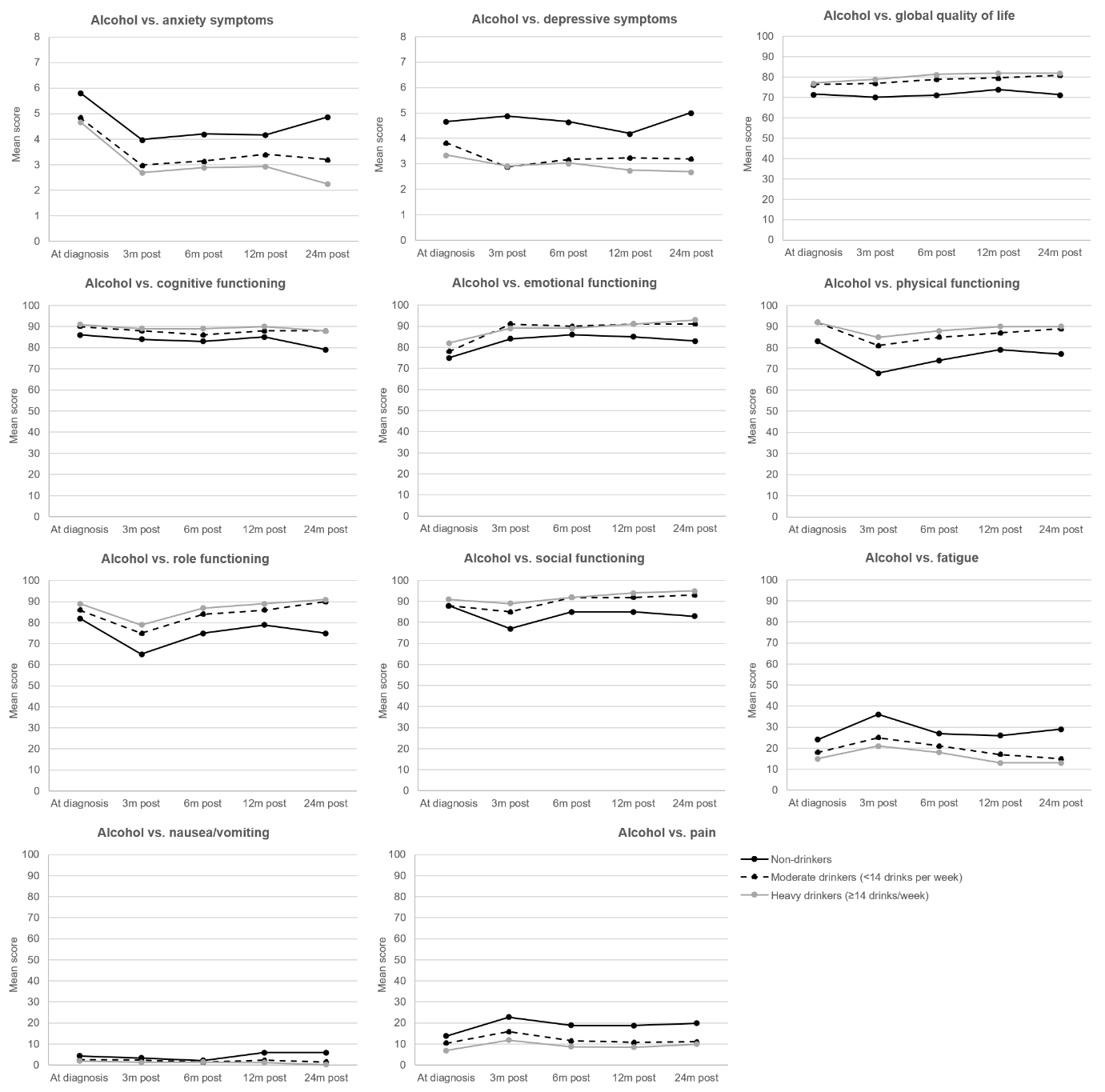


***Supplemental Figure 2****: Anxiety and depression, and all HRQoL domains and symptom scales are shown at each time point, and stratified for alcohol consumption categories (non-drinkers, moderate vs. heavy drinkers).*
